# Sham-Controlled Study of Optokinetic Stimuli as Treatment for Mal de Debarquement Syndrome

**DOI:** 10.3389/fneur.2018.00887

**Published:** 2018-10-25

**Authors:** Viviana Mucci, Tyché Perkisas, Steven Douglas Jillings, Vincent Van Rompaey, Angelique Van Ombergen, Erik Fransen, Luc Vereeck, Floris L. Wuyts, Paul H. Van de Heyning, Cherylea J. Browne

**Affiliations:** ^1^Department of Otorhinolaryngology and Head and Neck Surgery, Antwerp University Hospital, Antwerp, Belgium; ^2^Translational Neurosciences, Faculty of Medicine and Health Sciences, University of Antwerp, Antwerp, Belgium; ^3^Departments of Biomedical Physics, Faculty of Sciences, University of Antwerp, Antwerp, Belgium; ^4^Department of Rehabilitation Sciences and Physiotherapy, Faculty of Medicine and Health Science, University of Antwerp, Antwerp, Belgium; ^5^Multidisciplinary Motor Centre Antwerp (M^2^OCEAN), University of Antwerp, Antwerp, Belgium; ^6^School of Science and Health, Western Sydney University, Sydney, NSW, Australia; ^7^Translational Neuroscience Facility, School of Medical Sciences, University of New South Wales, Sydney, NSW, Australia

**Keywords:** Mal de debarquement syndrome, MdDS, optokinetic stimulation, placebo, spontaneous and motion triggered MdDS

## Abstract

**Introduction:** Mal de Debarquement Syndrome (MdDS) is a condition characterized by a perception of self-motion in the absence of a stimulus, with two onset types: Motion-Triggered and Spontaneous. Currently, the pathophysiology is unknown and consequently, the therapeutic options are limited. One proposed treatment protocol, developed by Dai and colleagues is based on optokinetic stimulation, which aims to re-adapt the vestibular ocular reflex. This study aimed to reproduce the treatment protocol developed by Dai and colleagues and to assess if a placebo effect is present in the treatment protocol and lastly, aimed to further investigate the treatment on MdDS patient outcomes.

**Method:** Twenty-five MdDS patients (13 Motion-Triggered and 12 Spontaneous) were exposed to 5 consecutive days of optokinetic treatment (consisting of exposure to optokinetic stimuli with head movements). Eleven of these 25 patients were also exposed to 2 days of a sham treatment prior to the OKN treatment. Posturography measurements and reported symptoms [e.g., using the visual analog scale (VAS)] of patients were assessed throughout the treatment. Posturography data of the patients was compared with the data of 20 healthy controls.

**Results:** No placebo effect was recorded with any changes in postural data and VAS scale. After the optokinetic treatment, a significant improvement in postural control was observed in 48% of patients, of whom 70% were of the Motion-Triggered subtype (*p*-values: Area under the Curve—Anterior Posterior < 0.001; Area under the Curve—Medio Lateral *p* < 0.001, Confidence Ellipse Area (CEA) < 0.001, Velocity < 0.001).

**Conclusion:** The protocol was effective in approximately half of the MdDS patients that took part in the study, with no placebo effect recorded. The Motion-Triggered group responded better to treatment than the Spontaneous group. In addition to this, this study indicates that the greatest postural changes occur within the first 3 days of treatment, suggesting that a shorter protocol is possible. Overall, these findings support what was previously observed in Dai's studies, that optokinetic stimulation can reduce and ease self-motion perception in those with MdDS. Thus, validating the reproducibility of this protocol, suggesting that a consistent and uncomplicated implementation across treatment centers is possible.

## Introduction

MdDS is a disorder characterized by a chronic perception of self-motion, often described as bobbing, rocking or swaying (1), which in most cases is accompanied by postural instability. Associated symptoms such as migraine, brain fog, secondary mood disorders, and cognitive fatigue are often reported by patients (2). Typically, MdDS is triggered by disembarking from a moving vehicle (e.g., a cruise, flight, car ride, etc.) (3) and is then defined as “Motion-Triggered (MT) MdDS” (4). However, the same symptoms can also appear spontaneously in individuals, in which it is defined as “Spontaneous (SO) MdDS” or “non-MT MdDS” (4, 5). The term “SO MdDS” has been used by others to describe patients with similar symptoms to MT MdDS patients, despite lacking both a motion stimulus and a “debarquement” event. Thus, defining these patients as having MdDS is technically not accurate in a literal sense, however the nomenclature has been used in this manuscript to allow for comparison with previous work (4).

In addition to this, the MdDS population (considering both onset subtypes) has a distinct female predominance, which has been widely described in several reports (2, 6–13). Furthermore, the average age of onset is generally between 40 and 50 years of age (7). Despite the growing awareness and investigations into MdDS, the knowledge of this condition among health care professionals is still limited, resulting in a high number of misdiagnosed patients (4) and poor treatment options. Among multiple hypotheses, one theory formulated by Dai and colleagues (1, 3), defines MdDS as the result of maladaptive coupling of multiplanar information of the vestibular-ocular reflex (VOR). This theory has been named as “maladaptation of the VOR” (3, 14, 15). Based on this theory, a treatment protocol was developed to readjust this maladaptation. The treatment uses optokinetic (OKN) stimulation (in the form of vertical and horizontal stripes) in combination with passive head roll movements to induce a “re-adaptation” of the VOR (1). It is known that in large visual fields, visual inputs have the ability to drive the VOR, which is called the optokinetic reflex (OKR), and this visual input can elicit a false perception of motion (16). In particular, Dai et al. (1) developed a personalized protocol, where head roll oscillation frequencies were matched to the patient's internal sensation of motion (rocking, swaying, etc.) and the direction of the OKN was usually customized and in the opposite direction to the self-motion perception of the subject. During OKN treatment, the researcher rolls the patient's head at ±20° according to their rocking or swaying frequency, while the subject passively watches moving stripes projected on a wall (1). The hypothesis and first trial was tested on 24 MdDS patients, 70% of which reported an improvement of symptoms (1). Following this initial investigation, the study was replicated in 2017 by Dai, including a larger number of patients: 120 MT and 21 SO MdDS patients (17); and a follow-up 1 year after treatment. One year after the treatment, the follow-up revealed that the success rate dropped from 78 to 52% in the MT group and 48% in the SO group (17). To date, no other studies on the MdDS OKN treatment has been performed, thus, in order to further validate this therapy for MdDS patients, additional scientific studies are necessary. Additional studies should provide evidence that the protocol is reproducible and therefore proving that it is a suitable therapy for MdDS patients (18).

This study intended to validate the reproducibility of the OKN treatment by performing a semi-standardized OKN protocol with similar methodology developed by Dai and colleagues. In this study, a semi-standardized protocol was created with the aim to simplify and assess new ways to implement the OKN treatment to MdDS patients. This study also aimed to assess for the first time if a placebo effect was present by using a control group for validating the observed postural changes (19). Overall, it was hypothesized that the protocol could be effective and reproducible, providing similar results to those recorded by Dai and colleagues, and that the treatment could not induce a placebo effect.

## Methodology

### Ethical approval, patient recruitment, and study population

Patients with MdDS were recruited during vertigo-specific consultations at the department of Otorhinolaryngology and Head and Neck Surgery at the University Hospital of Antwerp, Antwerp University, Belgium. Ethical approval was obtained through the Ethics Committee of Antwerp University Hospital, Antwerp, Belgium (IRB number 15/44/454). Each patient gave informed consent prior to the study. All investigations were conducted according to the principles expressed in the Declaration of Helsinki. Patients were diagnosed with MdDS, implementing the earlier published guidelines by our research group (4, 11). MdDS patients with SO onset were categorized using the early guidelines of our group (4) and were distinguished from Persistent Postural Perceptual Dizziness (PPPD) patients. The main feature that distinguishes those with SO MdDS and those with PPPD is that SO MdDS patients report a temporal alleviation of symptoms during the exposure to passive motion (e.g., being passenger in a car), whereas PPPD patients usually report aggravation or no improvement of symptoms in the same condition. The same number of patients in the study performed by Dai et al. (1) was replicated in this study, thus 25 patients were included. Only patients from the Euro zone (flight distance <5 h from Brussels, Belgium) were included in the study. The inclusion and exclusion criteria are reported in Table 1.

**Table 1:** Inclusion criteria based on the published updated guidelines (4, 11).

**Inclusion criteria**	**Exclusion criteria**
Patient with complaints of persistent (>1 month) Mal de Debarquement; reporting a prolonged sensation of self-motion (rocking, swaying and bobbing) after the exposure to passive motion, most frequently a boat trip, or travel over air or land	<18 years
Patients with MdDS symptoms, which occurred spontaneously or with atypical onset refer to (3) for details	Epilepsy diagnosis
Otoscopy and Video-nystagmography with normal results	Visual impairments that cannot be corrected with glasses or contact lenses
Tonal audiometry speech audiometry and tympanometry with normal results	Pregnant women
Standard MRI posterior fossa, performed pre-treatment with normal results	Female patients experiencing menstruation (on the days of treatment) due to a potential the aggravation of symptoms and increase motion sickness sensitivity (13, 20)
Electronystagmography (ENG), vestibular evoked myogenic potentials (VEMP) with normal results,	Patients taking antidepressant drugs [e.g., Selective serotonin reuptake inhibitors (SSRIs), Monoamine oxidase inhibitors (MAOIs), Tricyclic antidepressants, or benzodiazepines-however, these patients could be included if they ceased taking medication at least 1 week prior to the treatment week].
Patients whose complaints cannot be explained by another diagnosis.	

*Exclusion criteria based on clinical observations*.

### Research methodology

#### Questionnaires

Prior to the study, an intake questionnaire was given to the patients, which included epidemiological questions, as well as questions related to onset and symptom fluctuations (see Appendix 1 in Supplementary Material). Patients were also asked to complete a Misery Scale (MISC) questionnaire (21) each day of the treatment. Similarly, a Visual Analog Scale (VAS) questionnaire describing symptom severity (0 = no complaints, 10 = significant complaints) (21) was provided to the patient each day before and after the OKN stimulation. For more details, see Appendix 1 in Supplementary Material. In addition to the intake questionnaire, follow-up questions regarding symptoms 3 months post-treatment were presented (e.g., have your symptoms changed since the treatment? Have you experienced brain fog since the treatment?). For more details, see Appendix 2 in Supplementary Material.

#### Posturography

Posturography was measured with the use of a Wii Balance Board (Nintendo Co., Ltd), as it has been shown to be a valuable tool in measuring postural sway and the velocity rate of sways in vestibular patients (19, 22, 23) and similarly to what has been used in Dai's previous studies (1, 17). The data was analyzed with a program based on the Colorado University Wii Balance Board code developed at the Neuromechanics Laboratory at Colorado University (24). After acquisition, the data was filtered using a Butterworth filter of fourth order and a cut-off frequency of 0.17 Hz (MATLAB—Release 2017b, developed by The MathWorks, Inc., Natick, Massachusetts, United States). For the posturography measurements, patients were asked to stand upright for 1 min, with feet apart (hip width) on the Wii Balance board (19), with eyes closed, and barefoot (1). All patients remained on the board for a minimum of 1 min, although, if a patient had severe postural instability they could step off after 30 s. At all times, patients were closely observed by the researchers to avoid injuries such as falling off the board. Similarly to Dai and colleagues, a Wii Balance board was used, as this tool is inexpensive, portable and easy to be implemented, it has been regarded as a reliable tool to compare individual measurements and assess postural sway in several studies (19, 25–27). This measurement was performed before and after each exposure to the OKN stimuli as well as before and after the sham treatment. Eight posturography measurements were recorded every day before and after the exposure to sham treatment, and a range of 24–40 posturography measurements were performed over the 5 days of treatment. For details, see Table 2.

**Table 2:** Scheme of the stimulation performed throughout the sham and the OKN treatments.

	**Sham 1**	**Sham 2**	**Day 1**	**Day 2**	**Day 3**	**Day 4**	**Day 5**
Duration	4 min	4 min	4 min	4 min	4 min	Customized*	Customized*
Stripe movement	None	None	Vertical stripes, alternating right and left or vice versa	Vertical stripes, alternating right and left or vice versa	Vertical stripes, alternating right and left or vice versa	Customized*	Customized*
Head roll	0.165 Hz	0.165 Hz	0.165 Hz	0.165 Hz	0.165 Hz	Customized*	Customized*
	**Break**	**Break**	**Treatment end/Shorter treatment on Day 1**	**Break**	**Break**	**Break**	**Break**
Duration of OKN exposure	4min	4min		4min	4min	Customized*	Customized*
Stripe Movement	None	None		Vertical stripes, alternating right and left or vice versa	Vertical stripes, alternating right and left or vice versa	Customized*	Customized*
Head Roll	0.165 Hz	0.165 Hz		0.165 Hz	0.165 Hz	Customized*	Customized*
	**Break**	**Break**		**Break**	**Break**	**Break**	**Break**
Following sessions				Repeat as above	Repeat as above	Repeat as above	Repeat as above

*Patients who were not included in the sham group, started from Day 1 of the OKN treatment*.

* *Customized = If the patient responded well, the same protocol of Day 3 was used. If the patient did not respond well, the protocol would be customized*.

The following five postural parameter variables were recorded:

1. ***Area under curve Anterior-Posterior****(****AUC_AP****)*/2) ***Area under the curve Medial-Lateral (AUC_ML):***AUC_ML and AUC_AP represent the area under the curve of a power spectrum, assuming that patients suffering from MdDS reported aberrant medio-lateral sways, it is valid to assume that abnormal movements will result in aberrant frequencies of oscillation reported by the Area under the Curve in the power spectrum (28).
2. ***Confidence Ellipse Area ****(****CEA****)****:*** has been widely used before when assessing posture and this variable provides with 95% confidence ellipse area for the mean of the center of pressure (CoP) anterior, posterior, medial and lateral coordinates (25).
3. ***Path Length:*** the CoP path length (mm), which is the absolute length of the CoP path movements throughout the testing period (29).
4. ***Velocity :*** this variable is also named the *Sway Velocity*, representing the sway rate (mm/s) defined as the mean speed of movement of the CoP throughout the testing period (29).

**Figure 1:**
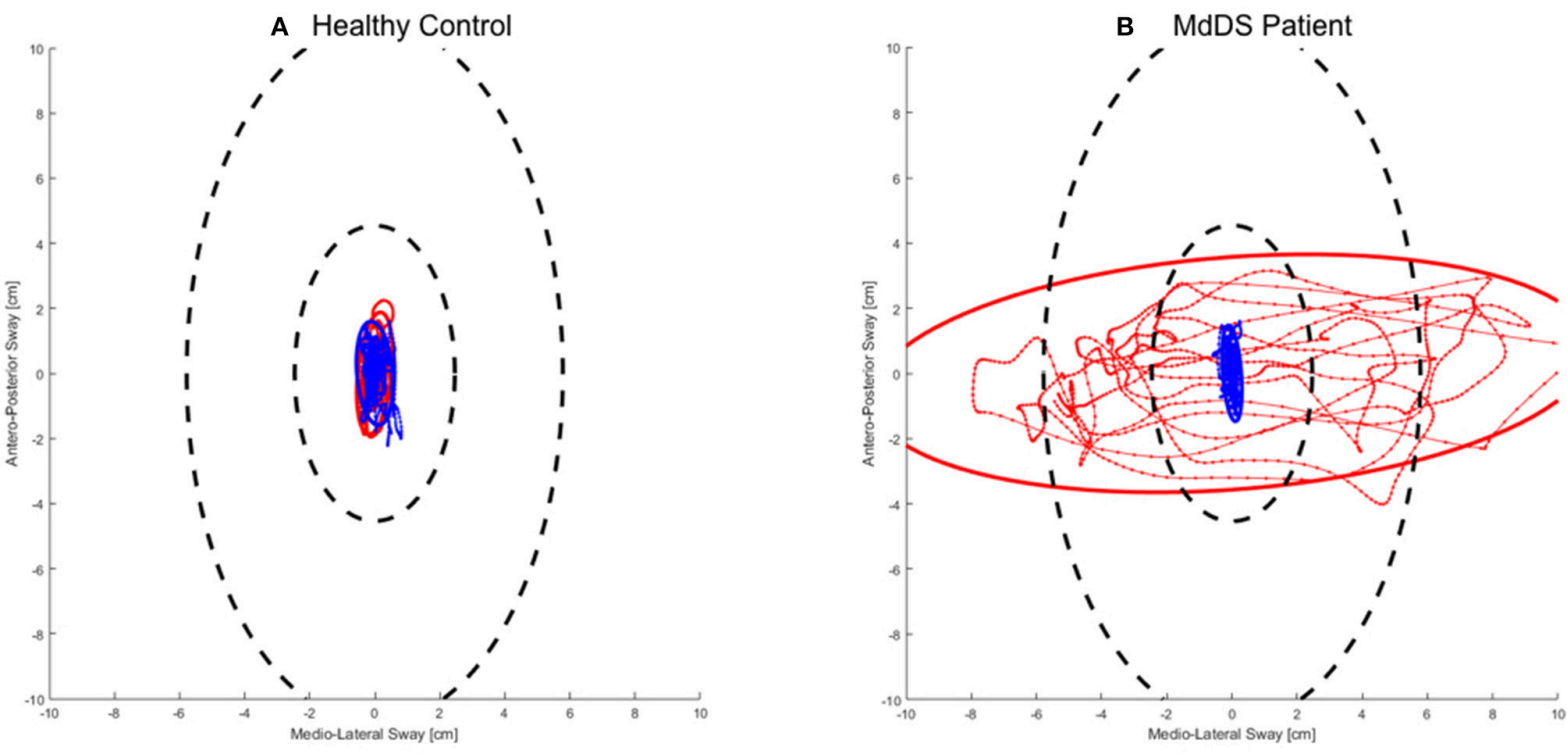
An example of the posturography recordings from a **(A)** Healthy control subject and **(B)** MdDS Patient – (Female/43 yr. old). The red lines represent the recording prior to the optokinetic treatment and the blue lines represent the recording after the optokinetic treatment for the patient and a test re-test for the healthy subject.

Twenty-five patients with MdDS were analyzed considering these five postural parameters. These parameters are hereafter referred to as “outcome parameters” for the remainder of the manuscript. The patients received the treatment over 5 consecutive days, and on each day, outcome parameter measurements were recorded before (Pre) and after (Post) treatment. An example of the posturography recordings of a healthy subject and a MdDS patient are shown in Figures 1A,B.

##### Posturography—control group

Twenty healthy control individuals were tested, using the test-re-test method, twice within a 2-week interval (on Day 1 and Day 2); however, they did not receive the OKN or the sham treatment. These subjects were age-matched (mean age: 44.5 years—ranging from 24 to 66 years) with the patient group. With this assessment we were able to evaluate whether any learning effects were present. However, the control subjects were not exposed to the Wii board as much as the patients who underwent the whole treatment (for the patients the exposure to the posturography measurements was ranging from 24 to 40 measurements).

#### Treatment (treatment and sham)

##### Optokinetic treatment duration

All patients underwent 5 consecutive days of OKN stimulation. During the 5 days of treatment, patients received various sessions per day: two sessions on Day 1, slowly increasing to 4 sessions on Day 2 and up to 6–8 or more sessions on Day 3. Thus, all patients underwent a gradual increase of exposure time and number of sessions throughout the 5 days with 30 min intervals between OKN exposures. On Day 4 and 5, the number of sessions were modulated according to their subjective perception of internal oscillation (e.g., if a patient was feeling better and their posturography improved, only 4 sessions were performed on Day 4 and Day 5, on the contrary if a patient's subjective perception and postural outcome measures were not improving, the patient's sessions were increased and customized).

##### Sham treatment duration

Among the 25 patients that were to receive the treatment OKN stimulation, 11 of those were semi-randomly selected for the Sham treatment, which involved two additional sham sessions prior to the OKN treatment. Patients were semi-randomly selected because the availability of the patients was taken into account. Participants were exposed to the same OKN stimulus, but without the stripes moving. They were asked to stare passively at static OKN vertical stripes, while their heads were rolled at the same frequency as the OKN treatment (0.165 Hz). It was hypothesized that introducing the patients to the same environment and being in contact with them (by holding and rotating their heads), this could potentially induce a placebo effect.

##### Optokinetic treatment setting

**Figure 2:**
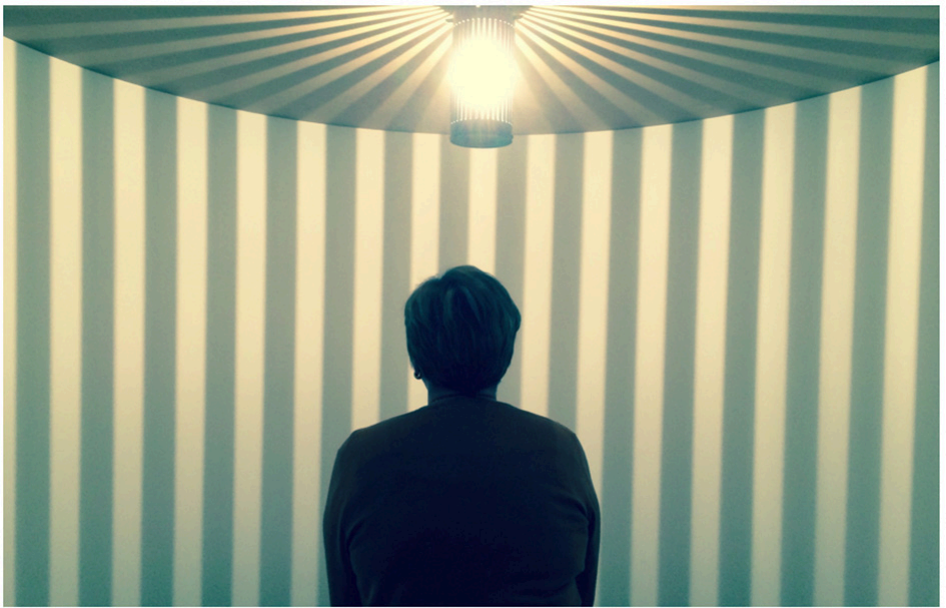
Laboratory Set-up showing full-field optokinetic stimuli as implemented in this study.

During the treatments, the patients were seated in a chair in a darkened OKN specific room, built for the experiment. A full-field OKN visual stimulus was projected on a semi-circular wall (Figure 2), which filled the whole peripheral visual field of the patient, similar to Dai's setting (17). Patients were seated 60cm from the wall and the semicircular wall had a diameter of 3 m [Field of View (FoV) = 71.56°, 1.249 rad]. During the treatment, the OKN stripes moved at a speed of 10°/s. The patients were instructed to stare passively at the stripes; they were instructed prior to the study on how to look straight in front of them without following the stripes movement with their eyes or starting at a fixated dot.

##### Head roll frequency

The patient's head was rolled at a constant frequency of 0.165 Hz by the researcher aided by a metronome. This frequency was chosen as it was considered the closest frequency to 0.167 Hz, a known frequency for emetic incidence (30), that was not able to induce any discomfort for the patients. This was different to what was done in Dai's protocol, where the head roll frequency was customized for each patient and the frequency was chosen according to the patient's perception of internal oscillation.

##### Direction of stripes

During the first 3 days, patients were only exposed to vertical OKN stripes moving right or left. The direction of the OKN stripes (e.g., stripes starting from the left or right first) was determined by two main variables—the Fukuda Stepping Test or the description of the patient's perception of internal oscillation considering the direction of the phantom motion (swaying, rocking, etc.). For the Fukuda Stepping Test, patients were asked to march in one spot for 45 s, with eyes closed and arms held out straight. An analysis was made of whether the patient rotated dominantly toward one side with a deviation >20° (1), as reported in the study of Dai and colleagues. The OKN stimulation was then programmed to start in the opposite direction to the one indicated by the results of the Fukuda Stepping Test. This was performed everyday before starting the trial. If an abnormal Fukuda Stepping Test was not observed in a patient, but they described a constant motion perception in one main direction, this variable was considered. In those cases, the OKN stimulation would start in the opposite direction to the patient's subjective perception. In this setting patients were systematically exposed to the opposite direction of the initial direction determined by the Fukuda Stepping Test, contrary to the customized approached proposed by Dai. This procedure was repeated throughout Day 1 and Day 3 and the same stimulation was gradually increased, meaning that it was repeated multiple times always including both directions. This is different from what proposed by Dai and colleagues were the OKN direction was also customized for each patient. For more details about the OKN sessions performed each day, see Table 2.

**Figure 3:**
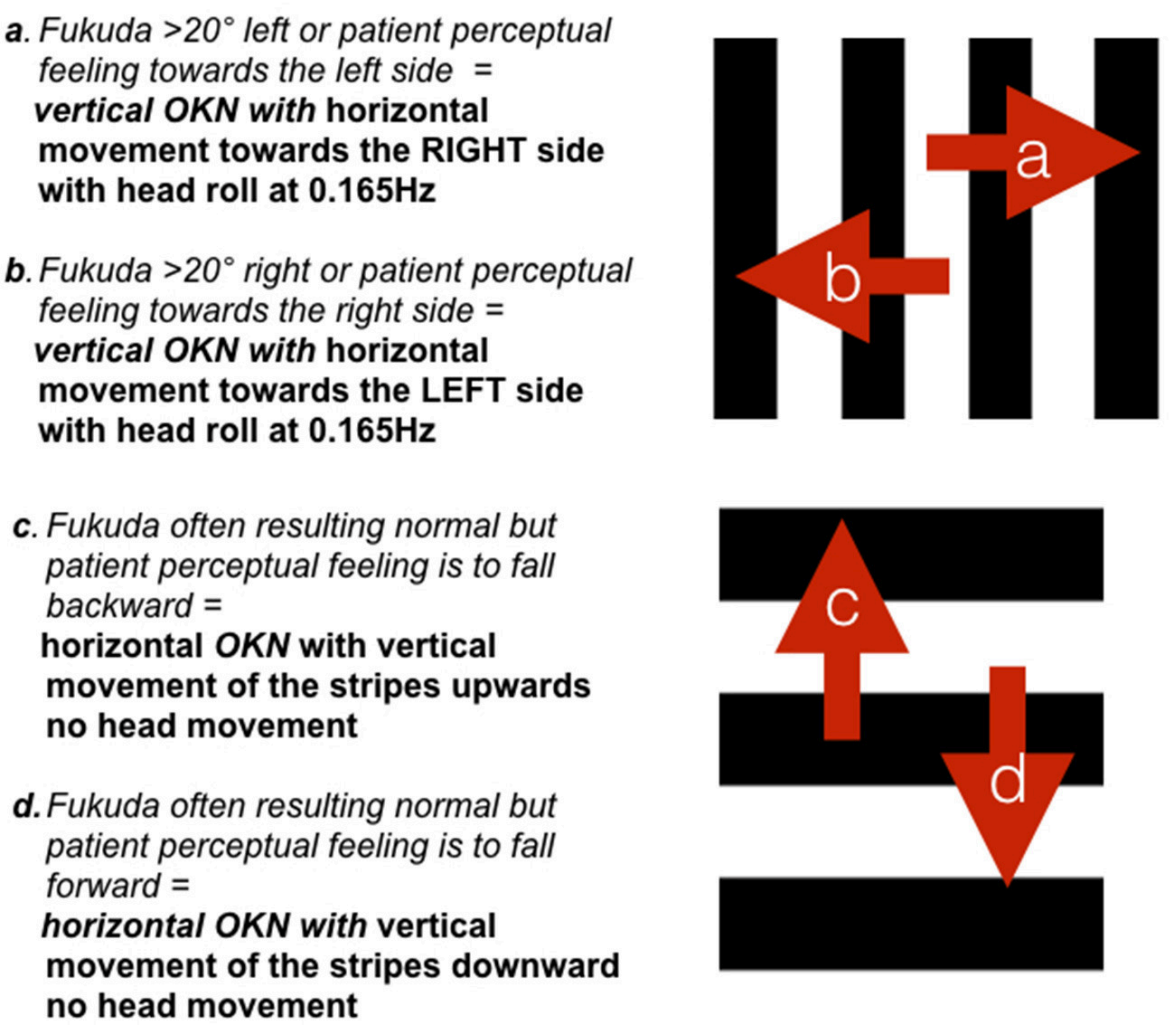
Schematic representation of the applied stripe movement according to the Fukuda Stepping Test results or the patient's perception of movement. Directions **(a,b)** were used during Day 1 to 3. Directions **(c,d)** were only implemented when required during the customized sessions on Day 4–5.

Following the first 3 days, where patients were only exposed to vertical OKN stripes moving right or left for 4 min per stimulation (see Figure 3 for stripe direction movements “a” and “b”), the OKN treatment protocol was customized for each patient if required. As a result, on Day 4 and 5, patients could be exposed to horizontal stripes moving upward or downward according to their subjective perception with a passive head roll at 0.165 Hz (see Figure 3: when patients subjective perception matched with “c” or “d”) if required. In those final days, if requested, horizontal stripes were used, however no head movements were performed during horizontal stripes and sessions lasted for 1 min. Only 10 patients were exposed to horizontal OKN stimuli with no head movement. Horizontal stripes were not implemented in the same room as for the horizontal stripes, but on a large flat screen, which was able to cover the whole patient's visual field. Patients were exposed to a reversed direction of the horizontal or vertical stripes if they reported a worsening of symptoms following one direction.

##### Sham treatment setting

After the posturography recording, the patients were seated on a chair in the same room as per the OKN treatment. During the sham treatment, the OKN stripes did not move and patients were required to stare passively (as described earlier) at the stationary stripes. The patient's head was rolled at 0.165 Hz frequency for 4 min, during which the researcher made use of a metronome to maintain this frequency. After this, the patient's posturography was measured for a second time. The patient was then re-exposed to the stripes for another 4 min with the same head movements. This was repeated in two sessions with 30 min interval between the two, with a total of four sessions over 2 days. For more details, see Table 2.

#### Statistical analysis

The responses to the questionnaires (for details, see Appendixes 1, 2 in Supplementary Material) were analyzed using Chi Square analysis, this was used when comparing different onsets. While when considering the group as a whole, the one-sample binomial tests analysis were performed. This analysis was performed on the questionnaires provided to the patients with regards to their symptoms (e.g., duration of onset, triggers, etc.). Four research questions (RQ) were made and here reported the statistical analysis applied.

##### RQ1: is the OKN treatment effective in reducing MdDS symptoms?

The difference between the *Pre-treatment* measurement and the first measurement of each day separately was tested using a linear mixed model, followed by a *post-hoc* analysis with Dunnett's Method for correction. This allowed us to observe if changes were retained until the morning after the last treatment, when each Pre-treatment measurement was taken, as well as to evaluate if the regular testing of the posturography induced an improvement. In addition, the Wilcoxon Signed-Rank Test (with Bonferroni correction) was used for the null hypothesis that within the day there was, on average, no change in postural outcomes within the subjects. This analysis allowed us to observe when the changes occurred within the 5 days of treatment. The success rate was defined as an improvement of postural stability when comparing the measurements recorded before and after the treatment. The patient needed to report an objective postural improvement, which is defined by a significant improvement in three of the postural outcome measurements. Postural outcome measures were interrelated and as a result, in most cases, when one changed, the others also showed a similar change. Together with this, a significant improvement in the patient's VAS score (3 points lower) and a statistically significant difference between Pre and Post VAS scores was required to qualify as treatment “success.” Patients were also asked to describe their subjective symptom perceptions. A 3-month follow-up questionnaire was also used to enquire patients about their symptom status after the treatment. In the follow-up, no posturography data was recorded, thus only reports of the subjective perceptions of the patients were collected. These two time points for success rate (i.e., end of treatment and 3 months follow–up) were compared using a McNemar's Test.

##### RQ2: does onset type, age, or gender and duration of symptoms (prior to treatment) influence the success rate?

To test if the difference in outcomes between measurement times was influenced by age, gender, and onset type, linear mixed models were used, with the individual as random intercept, fixed effects for time and resp. age, gender, and onset type, and the interaction between time and resp. age, gender, and onset time. The significance of this latter interaction term shows whether one of these three covariates has an effect on the change in outcome between the two measurement moments.

Specifically, regarding the duration of symptoms (prior to the treatment), an association between the duration of symptoms (i.e., duration onset) and success rate was investigated. Hereto, a Mann–Whitney *U*-Test was used to assess if the duration of the symptoms was significantly different between the groups.

##### RQ3: does the 2-day sham treatment involving a passive head roll at 0.165 Hz have a significant effect on patient posturography and on their subjective feeling of motion?

Using a Wilcoxon Signed-Rank Test, we tested if there was significant change in outcomes between measurement times within a patient in the Sham days (Bonferroni correction was applied); this non-parametric test was used due to the small group sizes.

##### RQ4: do postural data from patients differ from healthy controls that performed a test-re-test?

The healthy control group performed a test-re-test with a 2 week interval (Δ = measurement Day 2 Post—measurement Day 1 Pre), whereas for the MdDS patients, the interval was only 5 days, as the first day of treatment was considered as Day 1, regardless if patients had been exposed to 2 days of Sham (Δ = measurement Day 5 Post—measurement Day 1 Pre). A Mann–Whitney *U*-Test was used to test whether the Δ differed between cases and controls.

## Results

### Epidemiology—patient characterization

Patient epidemiological data, symptom duration and onset cause are reported in Table 3. All 25 patients reported that they generally experienced symptom fluctuations throughout the day (*p* < 0.01). Patients were asked if they felt more anxious since their MdDS onset, the great majority (84% MT-−75% of SO) reported to be more anxious since their onset (*p* = 0.003). All 25 patients reported to have had to modify their lifestyle after onset.

**Table 3:** Epidemiological data for Motion-Triggered and Spontaneous onset patients and cause of onset for Motion-Triggered patients only.

**Epidemiology**	**MT (n = 13) and SO (n = 12)**	
Mean age	42.3 years	
SD	11.3 years	
Female	17 (68%)	
Male	8 (32%)	
Symptom duration (mean)	3.9 years	
Symptom duration SD	4.5 years	
Symptom duration—Minimum	3 months	
Symptom duration—Maximum	19 years	
**Onset cause for MT patients**		
Cruise	4 (30%)	
Flight	7 (53%)	
Car ride	1 (7.6%)	
After a trampoline + train ride	1 (7.6%)	

### Treatment outcomes

#### RQ1: is the okn treatment effective in reducing MdDS symptoms?

Patient postural outcome measurements that were performed everyday prior to the daily treatment were compared with the first postural measurement of the following day. The relevant *p*-values are reported in Table 4.

**Table 4:** *p*-values from the linear mixed model test, testing the null hypothesis that the mean outcome does not differ between days. This was rejected considering the AUC_AP/AUC_ML; CEA and Velocity outcome postural variables.

**Postural parameters**	***p*-value**	***p*-value Bonferroni—corrected**
AUCap	4E-05	**2E-04**
AUCml	3E-04	**1E**-**03**
CEA	1E-05	**6E**-**05**
PathLength	3E-01	2E + 00
Velocity	5E-06	**2E**-**05**

*Statistically significant values are in bold text*.

Considering the results in Table 4 a *post-hoc* test using the Dunnett's Method for correction was performed (Table 5).

**Table 5:** *p*-values for the pairwise comparison of the measurements from Day 2, 3, 4 and 5, (obtained prior to the intervention) compared to the first postural measurement prior to the treatment (Day 1) (*posthoc* analysis with Dunnett's Method for correction).

	***p*-value**	***p*-value**	***p*-value**	***p*-value**
	**Δ = 2-1**	**Δ = 3-1**	**Δ = 4-1**	**Δ = 5-1**
AUC_AP	0.3705	**0.0289**	<**0.001**	<**0.001**
AUC_ML	0.464	**0.0184**	<**0.001**	<**0.001**
CEA	0.3449	**0.0064**	<**0.001**	<**0.001**
**PATHLENGTH**				
Velocity	**0.02247**	**0.00101**	<**0.001**	<**0.001**

*Statistically significant values are in bold text*.

**Figure 4:**
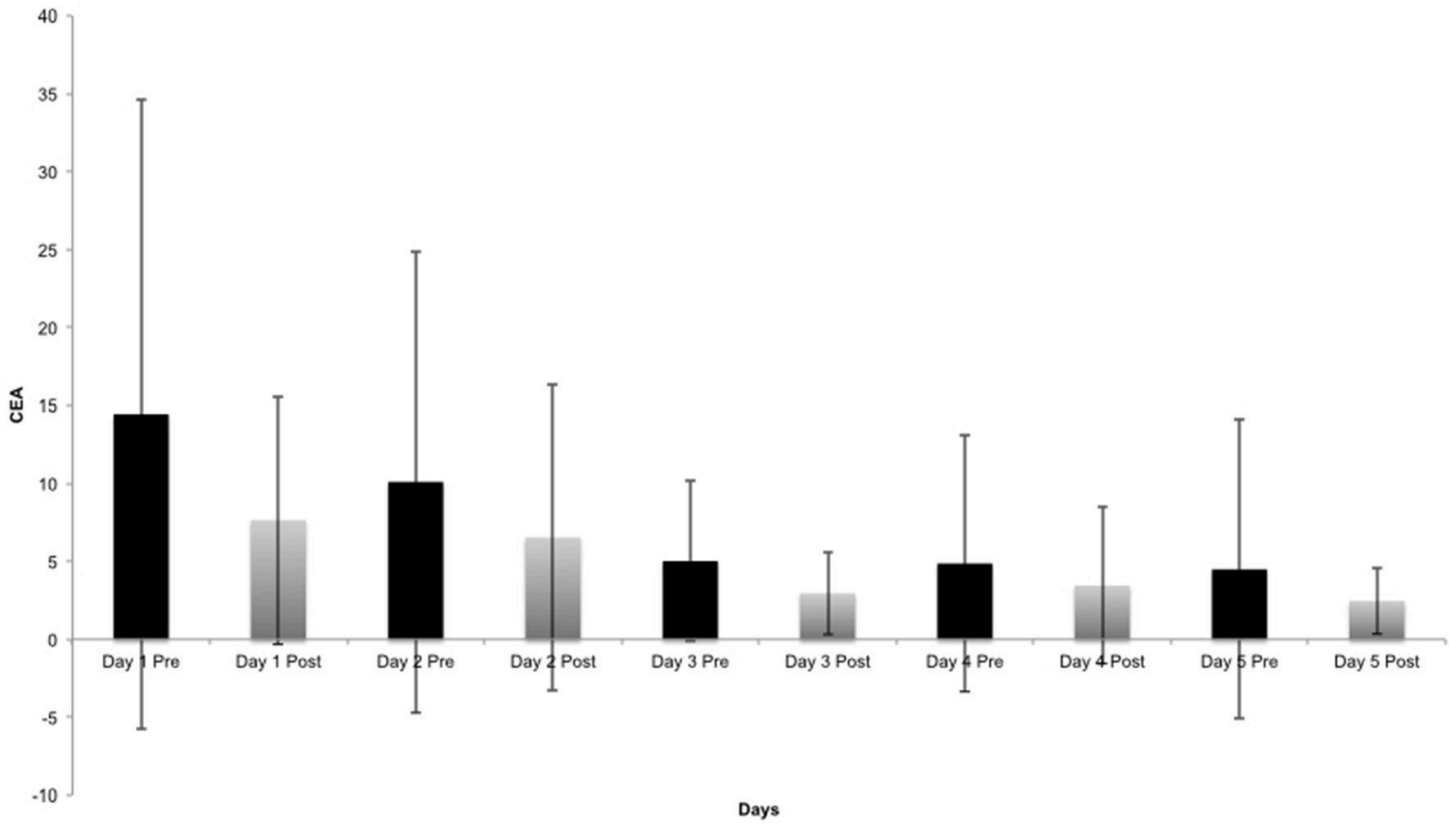
Breakout of CEA (95% confidence interval) changes, Pre and Post for each day, (considering the average of all patient's measurements).

Postural changes are detectable from the third day of treatment and the patient postural improvement remained significant throughout day 4 and 5 for AUC_ML, CEA, and Velocity. In addition to this, the immediate response of the treatment on the postural outcome measures was calculated, on each separate day. Figure 4 represents the differences (Pre-treatment—Post-treatment) in the Y-axis (CEA 95% confidence interval) vs. the treatment days in the X-axis.

#### The differences between pre- and post-measurements within the same day on day 4 and 5 show a small change occurred, compared to the pre-measurement on day 1 and day 2

The Wilcoxon Signed-Rank Test for the null hypothesis that during the course of 1 day, there is no change in postural outcome within the patients, resulted in a significant p-value for CEA outcome measure on Day 3 (*p* = 0.025) (Figure 4). Path length and Velocity also changed on Day 1 (*p*-values of 0.016 and 0.049, respectively). A large standard deviation among patients was reported on Day 1 considering CEA outcome, which decreases toward the end of the treatment. All *p*-values were corrected for testing five postural outcome measurements.

These posturography outcome measurements were in line with the subjective VAS questionnaire, where a reduction in the VAS score of minimum 3 points indicated a reduction in symptom severity. The VAS score reported a statistically significant change (*p* = 0.004) from Day 1 to Day 5, but Spearman correlations between posturography outcomes and VAS were very weak (data not shown). MISC questionnaire results did not differ significantly between baseline and the follow-up measurement.

#### RQ2: does onset type, age, or gender and duration of symptoms (prior to treatment) influence the success rate?

The rate of change between AUC_AP and AUC_ML did not differ between both two groups (MT and SO) and between the two genders (male and female), and it was not dependent on age (for details about the gender responses see Appendix 3, Figure b in Supplementary Material).

With regards to the *success rate*, the two onset (MT vs. SO) success rates were evaluated. The first success rate was noted on the last day of treatment and a second after a 3-month follow-up, patients were queried about their symptoms. Overall, 48% of the success rate was observed considering the last day of treatment, when considering the two onset subtypes separately, 70% of MT reported an improvement of symptoms. The same success rate (48%) was maintained after the 3-month follow-up. There was a significant association between both the last day of the treatment and the follow-up (*p* = 0.009, Chi-square test), but among the patients who reported a different result between the two success measurements, no trend was observed. The number of patients shifting from success to failure was not significantly different from the number of people shifting from failure to success (*p* > 0.05, McNemar's test). Specifically, three patients reported remission of symptoms in the 3-month follow-up, while in one patient, the phantom motion perception resolved, but severe migraine and brain fog were reported. Also a potential association between duration of symptoms and success rate was taken into account. No significant effect was found (*p* = 0.13, Mann–Whitney *U*-Test). Overall, SO patients had MdDS a median of 3 years and 7 months, while the MT patients reported MdDS symptoms with a median of 1 year and 7 months when they enrolled into the study.

#### RQ3: does the 2-day sham treatment involving a passive head roll at 0.165 Hz have a significant effect on patient posturography and on their subjective feeling of motion?

No significant differences were observed between the baseline measurement (prior to sham) and the last measurement following the second sham treatment when considering all the five outcome parameters (all *p* > 0.05, Wilcoxon Signed-Rank Test). Additionally, the subjective VAS score had a mean of 5.03 (SD = 2.08) on Day 1 of the sham and remained similar on Day 2 Post sham with an average score of 5.08 (SD = 2.44), as a result no significant differences in VAS score were observed.

#### RQ4: does postural data from patients differ from healthy controls that performed a test-re-test?

The fourth research question was to test if the postural changes between baseline (before starting the treatment on Day 1 of the treatment week) and Post-treatment (last recording collected after the treatment on Day 5) in the patient group were different from the test-re-test results in the healthy controls group.

The *p*-value obtained after testing for a difference in Δ between the cases and the healthy controls, indicates that patient postural changes were on average greater compared to healthy controls, with a significant effect in CEA and Velocity after Bonferroni correction for multiple hypothesis testing (Table 5). Sham days were here excluded.

### Symptoms reported in the follow-up

Within the follow-up questionnaire, patients were queried about their symptoms after 3 months following the treatment. A statistically significant difference was reported between SO and MT subtypes with regards to the follow-up symptoms (*p* = 0.010), with 70% of SO patients reporting the same level of symptoms as prior to the treatment while 7% of MT reported the same symptoms, matching with a higher success rate among the MT group. Within the MT subtype, 46% of the patients who benefitted from the treatment, meaning that their perception of motion greatly reduced or disappeared, still reported secondary symptoms after the OKN treatment, such as migraine, heightened visual motion sensitivity, brain fog, and headaches.

## Discussion

This study aimed to reproduce a similar protocol based on OKN intervention to ease MdDS symptoms as proposed by Dai and colleagues (1).

### Epidemiology—patient characterization

The patient group assessed in this study reflected the epidemiological description of previous findings. Firstly, a female predominance, as previously described (1, 2, 4, 7, 17), was also present in this study sample. The mean age of the patients was also similar to what has been previously described (mean 42.3 years), i.e., in the 5th life decade (2, 17). A similar number of MT and SO patients were randomly included. Equally, both onset subtypes reported to be greatly impacted by MdDS in terms of lifestyle, in line with previous research (4), in which MdDS was reported to have high levels of intrusiveness of an individuals life (31, 32). Patients were questioned about their symptomatology and in line with previous findings, symptom fluctuations throughout the day were reported by most patients (4). Additionally, they reported to be more anxious after MdDS onset, which is in line with previous research (33).

### Optokinetic stimulation and head roll as a treatment

During the treatment, patients reported an increase postural stability and a decrease in symptom severity, indicated by the VAS score results. These findings support earlier work by Dai and colleagues (1), i.e., that the patients' symptoms reduce significantly during the course of the treatment. Considering postural data, in order to observe whether changes were retained until the morning after each treatment day, Pre-treatment measures were compared. The most relevant changes were observed in AUC_AP, AUC_ML, CEA, and Velocity, indicating that patient's postural changes were retained until the day after. With a reduction in sway and sway velocity, it is theorized that patients became more stable due to the re-adaption of the VOR, as also discussed in Dai's study (1). In addition to this, a *post-hoc* analysis was performed, where each day was compared with the first initial postural measurement that was recorded. Significant postural changes occurred on the third day. This is particularly relevant, as it suggests that a shorter treatment protocol may be possible and that a standardized protocol is able to induce positive changes in MdDS patients. Further studies should consider comparing different treatment durations of the same protocol (e.g., 3 vs. 5 days). As reported in Table 6 the control group and patients groups were compared, highlighting that the CEA and Velocity parameter were statistically significant different.

**Table 6:** Postural outcome measurements Pre and Post treatment in patients and test re-test in the control group.

	**Control day 1 mean**	**SD**	**Control day 2 mean**	**SD**	**Patient pre day 1 mean**	**SD**	**Patient post day 5 mean**	**SD**	***p*-value (unadjusted)**	***p*-value (BF Corr.)**
AUC_ML	0.09	0.1	0.16	0.31	0.64	1.06	0.1	0.12	0.083	0.415
AUC_AP	0.51	0.36	0.42	0.35	1.51	1.8	−1.09	1.77		
CEA	3.61	3.12	4.19	5.77	14.44	20.18	2.47	2.12	0.002	**0.01**
Path Length	132.07	42.74	128.42	44.19	82.76	65.68	58.34	35.56	0.028	0.14
Velocity	2.16	0.7	2.08	0.74	1.71	0.99	1.05	0.4	0.006	**0.03**

*This table represents the mean and standard deviation (SD) of the case and control group at the two points of measurement. Significant p-values (bold) testing for a difference (using the Mann-Whitney U test) in test-re-test values between the two groups are reported. The last column shows the p-values after Bonferroni correction for 5 hypothesis tests. In bold text the statistical significant value. Abbreviations: AUC_AP, Area under curve Anterior-Posterior; AUC_ML, Area under the curve Medial-Lateral; CEA, Confidence Ellipse Area; BF Corr., Bonferroni Correction*.

When considering changes within each single day, large subject inter-variability is recorded in the first day of treatment (Figure 4), which decreases on the third day of treatment, suggesting that significant postural changes occurred after the first 2 days of OKN exposure.

Postural data of the patients was compared with data from a group of healthy controls, as suggested the literature (34), in order to identify any primary differences. No changes in postural outcome parameters were observed in healthy controls, as presented in the example reported in Figure 1. However, it has been recognized that the healthy controls were exposed to a much smaller number of posturography measurements than the MdDS patients; indicating that a potential learning effect could still be present. As such, a limitation to the study was the different time of exposure to posturography measurements between the patient and control group. However, from the data collected, considering the drastic changes over the days observed in the patient groups, it is hypothesized that the changes were genuinely attributable to the real OKN treatment involving the combination of OKN stimuli and head movements at the fixed frequency of 0.165 Hz. A control group that receive the same number of posturography measurements should be further explored. In addition to this, another limitation to this study was that after Day 3, when required, patients were exposed to customized stimuli, potentially affecting the integrity of a fully standardized protocol. Currently, it remains unclear what is the exact mechanism inducing these beneficial effects on MdDS patients, however, it is hypothesized that the VOR and the velocity storage are modulated by the OKN exposure and thus inducing a postural change by influencing the Vestibulo-Spinal reflex (VSR). In Dai's study from 2014 (1), it was hypothesized from previous studies on primates and humans that similarly MdDS patients may report an aberrant nystagmus and velocity storage integrator as a result to an adaptation to passive motion. Compensatory nystagmus was reported in an experimental model from NASA in 1962, where subjects in a rotation room were asked to roll their heads at different time intervals for 64 h (35). This study was one of the first to evidently remark the vestibular system's compensatory processes, for example when transferring to different environments (35). From these observations, Dai and colleagues developed a treatment using the full field OKN stimulus, which was directed toward normalizing the Velocity Storage Integrator back to its original orientation to gravity, i.e., the spatial vertical (3). This, according to Dai's protocol (1) involved rolling the patient's head at a customized frequency, while the subjects viewed a full field OKN stimulus moving in a direction opposite to a perceived vestibular imbalance measured by the Fukuda Stepping Test. In the current study, Dai's hypothesis was considered, and the first OKN exposure was always opposite to the direction of the Fukuda Step Test, however a different standardized protocol for the first 3 days was used. The OKN stimulus used moved slowly and generally produced a sensation of vection in the patients, this indicates that the OKN can activate the subcortical visual system that projects to the inferior olives, the vestibular nuclei, and the vestibulo-cerebellum (36). From the data collected, it can be concluded that most patients improved in the first 3 days of treatment, therefore when exposed to the standardized version. Additionally, no drastic improvements or postural changes were reported on Day 4 and 5, when patients could also be exposed to customized protocols, if needed. This suggests that there are no major differences between the two protocols, however future studies should consider comparing a customized protocol and standardized protocol in a larger cohort.

In addition to the Fukuda Step Test, Dai's protocol also examined the subject's nystagmus and used this parameter to adjust the OKN direction (1). A limitation to this study was that this test was not incorporated in the current study.

Further studies should investigate the presence of aberrant nystagmus in the MdDS population, for example, using Frenzel goggles (37).

While exposed to the OKN stimuli, the VOR responded to the stimuli with the activation of the OKR. The VOR and the OKR work together synergistically to maintain a stable retinal image, regardless of the type of motion one is subjected to (16). The VOR, as previously described, is a very fast acting reflex that serves to compensate for head movements in the 1–7 Hz range (38). As a result, however, it is much less accurate at lower frequencies. On the contrary, the OKR has opposite performance characteristics (38). The OKR has a longer latency due to the required evaluation of visual information to determine a response. Another relationship between VOR and OKR is related to VOR adaptation: it is known that the VOR response can adapt and accommodate sensory arrangements, as shown in a study by Draper (39), where VOR adaptation to virtual environments was tested. This study showed that the VOR response could modulate gain values to adjust the sensory re-arrangements occurring during stimulation. Taking this into account, Dai's theory can be further confirmed. Thus, the primary motion trigger of MdDS patients involved a head motion, while being subjected to different passive motion frequencies and an aberrant integration of visual and somatosensory stimuli may have resulted in the disruption of the VOR. A disrupted VOR leads to a disrupted velocity storage and VSR, which consequently leads to poor postural control. The results from this study supports Dai's theory that the OKN stimulation and head roll is able to induce a VOR adaptation process by altering the performance of the OKR through visual anomalies. The side-to-side (roll) head movements during vertical OKN stripes at 0.165 Hz frequency has proven to be effective for improving MdDS symptoms (sensation of self-motion, i.e., swaying, rocking). However, this is for now only a theory. Future testing is required to further assess if the changes in subjective perceptions of symptoms correlate with objective postural responses after the OKN treatment, for example by measuring the VOR gain or the optokinetic after nystagmus (OKAN) changes. One limitation to this study is that only postural changes induced by adaptations of the VSR in the form of posturography were measured, and that real VOR changes were not quantified.

When considering both onset subtypes, the success rate in this study was lower than what was reported in Dai's study (1). A 48% of the success rate (both MT and SO) on the last day of treatment was recorded in our setting, compared to the 78% reported by Dai's study (1), which could suggest that their personalized approach may be more beneficial for treatment success. Although, if closely analyzing and compare these two results, it is essential to consider that the study performed by Dai did not specify if SO patients were included, so it is presumed they only included MT patients. Thus, a more appropriate comparison should include only the MT patients from our cohort. When comparing Dai's findings with the data from the MT group in the current study, our success rate is similar to their findings (70% improvement in both studies). As such, it can be assumed that there are no major differences between the two protocols (3), but this requires further studies, as it remains unclear if the customization of the protocol is the primary responsible for the difference in success rate or if the inclusion of SO patients is also accountable for such difference.

Overall, when considering both onset subtypes, MT patients respond better to this type of treatment, with a higher success rate as compared to SO patients as reported in Dai's follow-up study (17), suggesting that this treatment may be more suitable for MT patients. From the results of this study, the SO patients had an average symptom duration that was slightly longer than the MT patients, with an average of 3 years and 3 months longer. However, in the cohort of this study, this variable did not significantly impact the success rate, when considering the two onset subtypes. The limited number of patients in this study does not allow us to conclude whether the duration of symptoms (from onset to treatment) can affect the success rate, thus we encourage future studies to enroll a larger number of patients and to consider the different onset subtypes.

With regard to the difference in treatment response, it should be noted that some SO patients did improve, as a result, this treatment should not be excluded from SO patients, but these patients should be aware of the lower success rate. This difference in success rate between the groups may suggest a potential difference in the underlying pathophysiology of the two onset types (5). It has been previously recognized that migraine affects more SO than MT patients and that most SO patients report to have been migraine sufferers before onset compared to MT (40), as a result it could be theorized that the pathological pathways for the SO group may be interrelated with migraine, and that this may be an important difference to the MT group.

The most recent studies on resting-state functional magnetic resonance imagining (rsfMRI) studies have shown an increased functional connectivity in MdDS patients between the left EC/amygdala and visual/vestibular processing areas, as a result of a decreased connectivity in multiple prefrontal areas (8). At this stage, it is unclear if the VOR maladaptation, which is a brainstem manifestation of MdDS, may also be implicated in a cortical manifestation with aberrant functional connectivity. It is possible that MT and SO patients share abnormal brain functioning and physiology, although acting via different pathways or mechanisms (40), which may explain why they respond differently to the OKN intervention. Also in line with Dai's findings (17), no differences among genders groups were observed. Both genders responded equally to the OKN stimulation.

### Follow-up

A series of follow-up questions were given to the patients 3 months after the treatment ended, where patients were able to report any fluctuations or changes in their symptoms since the end of the treatment. A greater number of SO patients reported the same symptom type (constant sensation of motion) and the same symptom level upon follow-up; this correlates with the poor success rate observed in this group from Dai's and colleagues findings (17). For the MT patients, the level of symptoms reported at the follow-up was much lower, indicating that the OKN treatment was able to reduce their MdDS symptoms up to 3 months follow-up. However, despite a general improvement in postural stability and self-perception of internal oscillation, MdDS patients from both onset subtypes still reported other associated symptoms such as migraine, and visual motion sensitivity, which prevented us from defining them as completely “symptom-free.” Further research and additional therapeutic approach should closely evaluate the overlap of migraine and high visual motion sensitivity symptoms in MdDS patients. Given these additional features, it can be argued that a more complex neural basis may be implicated in the pathophysiology of MdDS (41). A clear limitation to the current study is the relatively small sample size and the short-term follow-up. Therefore, further testing with a larger sample of patients and continuous follow-up to up to 2 years after the treatment is encouraged.

### Placebo study

The current study has also demonstrated that the sham treatment performed on MdDS patients from both onset subtypes does not induce a placebo effect. If a placebo effect were to occur, postural improvements after 2 days of sham sessions would be expected. No significant posturography changes were observed after the exposure to the sham treatment. This supports that the results acquired during treatment showed a legitimate improvement in postural stability and that sham was not able to induce any changes. Moreover, the subjective perceptions as reported by the VAS results did not change during the sham treatment, nor improve during the sham treatment days, indicating that patients' symptoms were neither aggravated nor improved. It was hypothesized that if patients were susceptible to a placebo effect, the interaction with the researchers and being enrolled in the study could have influenced their posturography outcome measures and subjective perception of internal oscillation, however it appears that this hypothesis can be rejected.

## Conclusion

This study was the first to reproduce and validate an OKN treatment in MdDS patients, similar to the protocol developed by Dai and colleagues, and the first to implement a standardized version of the treatment protocol. The current study has demonstrated that a placebo effect was not induced in patients exposed to the OKN treatment with passive head roll. Secondly, this study has shown that a gradual and standardized OKN treatment (OKN stimuli in combination with a head roll at 0.165 Hz) was able to reduce MdDS symptom levels in almost half of the MdDS patients involved in the study, and that MT patients benefitted the most from the treatment. The latter suggests a potential difference in pathophysiology in MT and SO MdDS; however, future studies should directly assess this. With this study, it has been shown that the OKN treatment can lead to an almost immediate postural improvement in MdDS suffers, within the first few days. As such, a shorter and more refined protocol (with head roll maintained at 0.165 Hz) may be beneficial and effective for MdDS patients. Future studies should consider a larger sample size and should investigate if these improvements are long term, and have an explicit comparison of different frequencies and OKN stimuli directions within the same study. The comparison of customized vs. standardized protocols requires further testing. Overall, the results described in this study showed that the OKN protocol developed by Dai and colleagues is reproducible. Thus, se hope that our data provide a basis for further research and that this treatment will be implemented across multiple treatment centers, as it is currently one of the most successful treatments for MdDS.

## Author contributions

VM is the primary author and was involved in the study design, patient recruitment, data recording and analysis and the writing of the manuscript. TP was involved in the data acquisition and analysis. SJ was involved in the data acquisition. VVR was involved in patient recruitment and supervision of the medical condition of the patients. AVO was involved in the study design and writing of the manuscript. EF was supporting the team with data analysis and statistical modelling. LV was involved in the design of the project and in the selection of the control group. FLW helped in the creation of the protocol, study design, data acquisition, data analysis and in the writing of the manuscript. PVH supervised the whole study and contributed to the implementation of the study and patient management. CJB was involved in the supervision of the study and in writing process of the manuscript.

### Conflict of interest statement

The authors declare that the research was conducted in the absence of any commercial or financial relationships that could be construed as a potential conflict of interest.
