# Supplementary material for: Sham-Controlled Study of Optokinetic Stimuli as Treatment for Mal de Debarquement Syndrome
Source: Front Neurol. 2018 Oct 25;9:887. doi: 10.3389/fneur.2018.00887 (PMC6210740; doi:10.3389/fneur.2018.00887)
Supplement: Supplementary file 1 [file Data_Sheet_1.docx]

**Supplementary Material / Appendix:**

**Sham-controlled study of optokinetic stimuli as treatment for Mal de Debarquement Syndrome**

Mucci, V.^1,2,3^, Perkisas, T.^3^, Jillings, S. ^2,3^, Van Rompaey, V.^1,2^, Van Ombergen, A.^1,2,3^, Fransen, E.^3^, Vereeck, L. ^4,5^, Wuyts, F.L.^3^ Van de Heyning, P.H.^1,2,5^, Browne, C.J.^6,^

***Corresponding Author:**

Viviana Mucci: [viviana.mucci@gmail.com](mailto:viviana.mucci@gmail.com) / [viviana.mucci@uantwerpen.be](mailto:viviana.mucci@uantwerpen.be)

**Appendix 1:**

**General Intake Information**

1. Name:
2. Gender:
3. Age:
4. When were you diagnosed with MdDS?

**MdDS Symptoms**

1. To the best of your knowledge, what was the motion event that induced your MdDS (e.g., cruising, boating, air flight, train)? *(Please specify onset date and if MdDS occurred spontaneously).*
2. Are your symptoms persistent or do your symptoms change according to different days? *(Describe the fluctuations and if the changes are related to particular events: stress, menstrual cycle)*
3. Do you feel more anxious since your MdDS appeared? *Yes / No*
4. Have you undergone Vestibular Rehabilitation for MdDS? *Yes / No* (If Yes, please specify)

**Lifestyle**

1. Does your MdDS affect your employment? *Yes / No*
2. How much does MdDS impair your lifestyle? *(Mention something you were able to do and now you have stopped or have difficulties in doing now)*

**Medications to ease MdDS Symptoms**

1. List your current medications, why you take them, date you started them, response, and side effects (if any). Please specify what you have been given for MdDS symptoms.
2. List any past medications you were given for MdDS

**Misery Scale Questionnaire** from**:** Bos JE, MacKinnon SN PA (2005) Motion sickness symptoms in a ship motion simulator: effects of inside, outside, and no view. Med, Aviat Space Environ 76:111–8

**Visual Analogue Scale:**

How would you describe your symptoms?

 No complaints Significant complaints

**Appendix 2**

**Follow up symptoms**

1. Please state how your symptoms have changed (improved or become worse) since the treatment?
2. Since the treatment, do you feel dizzy when watching busy traffic intersections, scrolling on your phone, walking in a supermarket?
3. Have you experienced brain fog since the treatment?
4. Have you experienced headaches since the treatment?
5. Have you experienced a migraine since the treatment?

**Appendix 3**

*
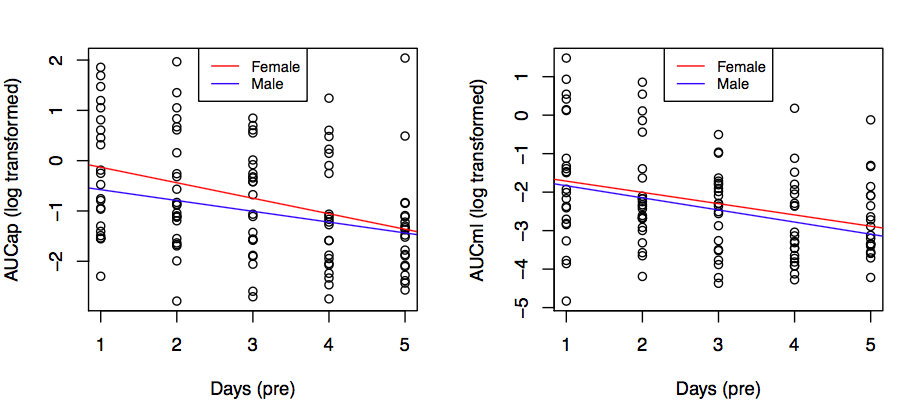
*

***Figure b:*** *AUC_AP and AUC_ML for female and male MdDS patients over 5 days of treatment, the graphs report an equal postural adjustment throughout the days.* No gender differences were reported. Abbreviations: AUC_AP= Area under the Curve Anterior – Posterior; AUC_ML= Area under the Curve Medio-Lateral

As report in Figure b, female (represented in red) and male patients (represented in blue) responded similarly in response to the optokinetic stimulation.
